# Ketamine as a Novel Psychopharmacotherapy for Eating Disorders: Evidence and Future Directions

**DOI:** 10.3390/brainsci12030382

**Published:** 2022-03-12

**Authors:** Anya Ragnhildstveit, Matthew Slayton, Laura Kate Jackson, Madeline Brendle, Sachin Ahuja, Willis Holle, Claire Moore, Kellie Sollars, Paul Seli, Reid Robison

**Affiliations:** 1 Department of Psychology and Neuroscience, Duke University, Durham, NC 27708, USA; matthew.slayton@duke.edu (M.S.); paul.seli@duke.edu (P.S.); 2 Integrated Research Literacy Group, Draper, UT 84020, USA; ljackson@cedarpsychiatry.com (L.K.J.); mbrendle@cedarclinicalresearch.com (M.B.); willis.holle@irlg.org (W.H.); 3 Novamind, Draper, UT 84020, USA; sachin@novamind.ca (S.A.); claire@novamind.ca (C.M.); ksollars@cedarpsychiatry.com (K.S.); reid@novamind.ca (R.R.); 4 Department of Pharmacotherapy, University of Utah College of Pharmacy, Salt Lake City, UT 84112, USA; 5 Department of Psychiatry, University of Utah School of Medicine, Salt Lake City, UT 84108, USA

**Keywords:** esketamine, ketamine, ketamine-assisted psychotherapy, eating disorder, anorexia nervosa, bulimia nervosa, binge eating disorder, pharmacology, psychedelics, treatment

## Abstract

Eating disorders (EDs) are serious, life-threatening psychiatric conditions associated with physical and psychosocial impairment, as well as high morbidity and mortality. Given the chronic refractory nature of EDs and the paucity of evidence-based treatments, there is a pressing need to identify novel approaches for this population. The noncompetitive N-methyl-D-aspartate receptor (NMDAr) antagonist, ketamine, has recently been approved for treatment-resistant depression, exerting rapid and robust antidepressant effects. It is now being investigated for several new indications, including obsessive–compulsive, post-traumatic, and substance use disorder, and shows transdiagnostic potential for EDs, particularly among clinical nonresponders. Hence, the aim of this review is to examine contemporary findings on the treatment of EDs with ketamine, whether used as a primary, adjunctive, or combination psychopharmacotherapy. Avenues for future research are also discussed. Overall, results are encouraging and point to therapeutic value; however, are limited to case series and reports on anorexia nervosa. Further empirical research is thus needed to explore ketamine efficacy across ED subgroups, establish safety profiles and optimize dosing, and develop theory-driven, targeted treatment strategies at the individual patient level.

## 1. Introduction

Eating disorders (EDs) are highly prevalent, disabling, and potentially fatal psychiatric illnesses characterized by abnormal eating and weight disturbances [1,2]. They are etiologically complex and multifactorial in nature, often leading to severe psychological and somatic complications [3,4,5,6], marked functional impairment [7,8,9], and poor quality of life and overall prognosis [10,11,12]. The socioeconomic burden of EDs in the United States is estimated to be at USD 64.7 billion annually, equating to USD 11,808 per individual diagnosed [13]. Nonfinancial yet significant reduction in personal well-being is further valued at USD 326.5 billion [13]. Approximately 8–15% of children and adolescents [3,14] and 16% of adults [15] are affected by EDs, with weighted population means of lifetime prevalence at 1.6% for anorexia nervosa (AN: 1.4% women, 0.2% men), 2.5% for bulimia nervosa (BN: 1.9% women, 0.6% men), 3.8% for binge eating disorder (BED: 2.8% women, 1.0% men), and 7.9% for eating disorder not otherwise specified (EDNOS: 4.3% women, 3.6% men) [16]. However, the prevalence of EDs is greatly underestimated, primarily due to variable diagnostic classifications, underreporting, and lack of research funding [17]. Moreover, EDs are associated with significantly elevated morbidity and mortality, compared with the general population [18,19], with the highest rates occurring in AN (standardized mortality ratio (SMR) = 5.9–10.5) [20,21]. Anorexia nervosa, in particular, carries a 12-fold increased risk of death—higher than any other psychological condition—to which low body mass index, poor social adjustment, and alcohol dependence have been reported as significant predictors [22]. Mortality rates for EDs are further complicated by concomitant psychiatric comorbidities (e.g., anxiety, depression, and substance abuse), as well as symptom persistence (12 months).

While various models have attempted to explain ED pathogenesis, the mechanisms subserving disease onset, progression, and maintenance remain not fully understood [23]. Notwithstanding, several hypotheses surround the neurobiology of EDs, which is supported by a growing body of literature (reviewed in Frank et al. [24] and Finch et al. [25]). Studies over the past decade have predominantly focused on correlating dimensions of ED pathology with structural brain changes, specifically in gray matter volume (GMV) and cortical thickness (CT). Evidence suggests that restrained eating is associated with aberrant activity in a complex network of regions implicated in emotional, self-related, and visuospatial processing, and is linked to increased GMV in the parietal lobule, precuneus, and parahippocampal, temporal, and frontal gyri [25]. These areas have fundamental roles in regulating affect, interpreting social information, and integrating spatial cues. Impairments may consequently reinforce restrictive cognitions and behaviors, in addition to body image distortions, present in AN. Comparatively, disinhibited eating is associated with altered activation in regions implicated in reward, emotional, and motivational processing, and is linked to increased GMV in the orbitofrontal gyrus, an area responsible for encoding value representations of food, as well as decreased GMV in the cingulate cortex, an area critical for regulating affect and goal-directed behavior [25]. Hyperactive reward- and hedonic-based responses to food may therefore drive overconsumption present in BN and BED. Evidence on CT, however, is heterogeneous across ED studies, with varying reports of high or low CT that persists or normalizes after recovery [26]. This discrepancy may in part arise from variation in neuroimaging and analytic techniques, suggesting that standard approaches are needed to reliably define ED pathophysiology and subgroups [27,28]. Studies have further investigated functional brain changes in EDs, identifying several intrinsic connectivity networks that may underlie and perpetuate maladaptive eating, including the executive control network (ECN) [29,30,31,32,33,34,35], default mode network (DMN) [30,35,36,37,38], reward network (RN) [29,39,40,41,42,43], and salience network (SN) [35,44,45,46]. These circuits are involved in goal-directed attention and cognitive flexibility, mentalizing and interoception, reward processing and sensitivity, and salient stimuli detection, respectively. As such, impairments may perturb adaptive eating behavior and body perception, as well as food reward valuation and readiness. However, data remain limited by the number of available studies, small sample size, and inconsistent methodology, and should be considered with caution. Evidence on functional network involvement beyond AN and BN is additionally lacking [47].

Currently, the primary care pathway for EDs is psychological and dietetic intervention, followed by psychotropic medication [48]. Treatment is generally provided on an outpatient basis, with medically compromised individuals recommended to higher levels of ED care, including intensive outpatient, partial hospitalization, and residential programs [49,50]. For BN [51,52] and BED [53], cognitive behavioral therapy (CBT, transdiagnostic) remains the most effective psychological treatment in managing ED symptomatology; however, such efficacy is not established for AN [54]. Other evidence-based modalities shown to yield clinical benefit for EDs include focal psychodynamic therapy (FPT, psychodynamic) [55,56], specialist supportive clinical management (SSCM, atheoretical) [57,58,59], and the Maudsley model of anorexia nervosa treatment for adults (MANTRA, cognitive–interpersonal) [59,60]. Family-based treatment (FBT, atheoretical) is particularly effective for children and adolescents [61,62,63]. Dietetic interventions are additionally used in the management of EDs and are common adjuncts to psychotherapies. Such interventions are aimed at normalizing eating behavior, achieving calibrated weight restoration and healthy weight management, and providing nutritional guidance necessary for sustained recovery [64,65]. While dietetic approaches are highly variable, they often include fear exposure to high-energy, palatable foods; increased dietary intake of fats, oils, and calcium; and enculturated practice of preparing food, sizing portions, and eating socially. However, insufficient data support the assessment, implementation, and efficacy of dietetic interventions for ED patients [66]. Recognition of this gap has prompted new research in the field, including studies on ethyl-eicosapentaenoic acid (ethyl-EPA) supplementation [67,68], as well as development of evidence-based practice guidelines [69,70,71,72]. In regard to second-line pharmacotherapies for EDs, drug classes comprise antidepressants (e.g., fluoxetine, citalopram, fluvoxamine, and sertraline), antiepileptics (e.g., carbamazepine and topiramate), opioid antagonists (e.g., naltrexone, naloxone, and nalmefene), and neurostimulants (e.g., lisdexamfetamine). These psychotropics are principally used to treat BN [73,74,75] and BED [73,75,76], showing modest improvements in impulse control (i.e., regulation of binge eating and purging), cognitive distortions (e.g., dichotomous thinking and catastrophizing), and concomitant psychiatric comorbidities (e.g., anxiety and depression). Despite no approved pharmacological agents available for AN, atypical antipsychotics—namely, olanzapine [77,78] and aripiprazole [79]—have been used to augment weight gain and reduce ritualistic tendencies around food. However, clinical management of AN, by and large, remains difficult, with limited and discouraging data supporting psychotherapeutic approaches [80] and medication trials [81]. Poor management of AN is reflected by disease chronicity and low remission rates [82].

Relatedly, three studies evaluating long-term trajectories of EDs found that 64% of patients with AN (*N* = 1693) [83], 53% of patients with BN (*N* = 2033) [84], and 30% of patients with BED (*N* = 68) [85] met full diagnostic criteria for an ED at 10–20 years follow-up. Outcome predictors included symptom severity (AN), illness duration (AN), psychiatric comorbidity (BN, BED), treatment age (BN), follow-up length (AN, BN), global functioning (BN), body dissatisfaction (BED), drive for thinness (BN), impulsivity (BED), sexual abuse (BED), and self-injury (BN), respectively. More than 50 years of literature on EDs further suggests that less than half of sufferers achieve full remission, a third experience residual symptoms, and a fifth become chronically ill [86,87,88]. Individuals with repeat treatment failures over protracted periods are considered to have severe and enduring EDs, with active disease cutoff points typically set at seven years in duration [89,90]. However, thresholds for severe and enduring EDs vary between studies (e.g., 5–10 years) due to the lack of an empirically derived and -accepted definition in the field [89,91]. “Both the clinician and [chronically ill] patient often share the experience of hopelessness and despair about the likelihood of meaningful change” [92]. Given the chronic refractory nature, increased risk of premature death, and paucity of high-quality, evidence-based treatments associated with severe and enduring EDs, pragmatic shifts toward harm reduction, palliative care, and quality of life *over* recovery have been proposed for this subpopulation [58,89,92,93,94,95,96]. This stems from efforts to minimize adverse impacts on sufferers, their caregivers and external support systems, and society at large [82,97].

Ketamine, an N-methyl-D-aspartate receptor (NMDAr) antagonist, is a dissociative anesthetic used for diagnostic and surgical procedures, as well as peri- and postoperative pain management [98,99]. It was developed in the 1960s as a fast-acting alternative to phencyclidine (PCP) [100] and is a 1:1 racemic mixture of its two optic enantiomers: S(+)-ketamine (esketamine) and R(-)-ketamine (arketamine) [101,102]. Comparatively, S(+)-ketamine has a 3–4 fold higher binding affinity for the NMDA receptor than R(-)-ketamine [103] and carries stronger anesthetic and analgesic potency [104,105,106]. Ketamine is further known to produce psychotomimetic and psychodysleptic side effects (e.g., depersonalization and derealization) in addition to ephemeral increases in glutamate release, with downstream activation of brain-derived neurotrophic factor (BDNF) and mechanistic target of rapamycin (mTOR) signaling pathways to promote synaptogenesis and neuroplasticity [107]. In 2000, the first double-blind, placebo-controlled trial of ketamine in depressed patients revealed that subanesthetic doses (0.5 mg/kg infused over 40 min) had rapid and robust antidepressant effects [108,109]. Numerous studies have since replicated this finding [110,111,112,113,114], with meta-analyses showing acute and prolonged antidepressant efficacy of single and repeated ketamine administration [115,116,117,118]. Other studies have described synergistic therapeutic effects when ketamine is administered as an anesthetic adjunct in electroconvulsive therapy (ECT): the gold standard for treating refractory depression [119,120,121,122]. While data remain varied, ketamine may be an effective treatment alternative to ECT altogether, with reports of faster antidepressant action and improved neurocognitive performance, specifically in attention, memory, and executive functions [123,124,125]. This eventually led to the approval of intranasal esketamine (Spravato^®^) for treatment-resistant depression (TRD) by the Food and Drug Administration (FDA) in 2019 [126,127]. Recently, ketamine has been investigated for several new indications [128,129], including obsessive–compulsive [130], post-traumatic [131,132], and substance use [130,133] disorder. The interest in using ketamine in EDs originates from (1) its capacity to reduce cognitive, affective, and behavioral symptoms among psychiatric nonresponders [134,135], and (2) the pressing need to identify treatment alternatives for EDs, of which are increasingly prevalent [16], have yet to benefit from pharmacological progress [73], and remain a leading public health concern [12].

## 2. Use of Ketamine in Eating Disorders

To date, few studies have examined the therapeutic use of ketamine for EDs, which are limited to case series [136,137] and reports [138,139,140] (Table 1), and are focused on AN over other primary (BN and BED) and secondary (pica, RD, and ARFID) subgroups. Mills et al. first introduced the dissociative anesthetic as a novel treatment for compulsive EDs in 1998, in a study where 15 patients with atypical, chronic refractory AN of 11.3 years ± 5.0 were treated with intermittent ketamine infusions combined with oral nalmefene, an opiate receptor antagonist [136]. In particular, AN presentation consisted of no comorbidity (*n* = 5), BN comorbidity (*n* = 2), OCD comorbidity (*n* = 5), BN and OCD multicomorbidity (*n* = 1), and BN and AUD multicomorbidity (*n* = 2). Patients received 2–15 ketamine infusions scheduled at 5–21-day intervals, dependent upon clinical response, and were delivered at 20 mg/h over 10 h. This was a fairly intense drug regimen—relative to current studies on ketamine and mental health [141] previously used to treat postoperative pain [142] and acute war injuries [143]. Marked and sustained remissions were observed in responders (*n* = 9) compared to nonresponders (*n* = 6), with no-to-minimal disease activity at 7–24 months follow-up. Moreover, responders showed significant reductions in obsessive–compulsive neurosis (*p* 0.001), in addition to increased weight acceptance, partial-to-complete weight restoration, and resolved amenorrhea. No significant improvements were reported for nonresponders. Investigators attributed this result to premature relapses following treatment, during which compulsive drives may have been reestablished, and/or the result of insufficient doses of nalmefene. Overall, clinical response (≥50% reduction in symptom severity) was associated with AN subtype (-R, restricting; -BP, binge/purge).

A recent longitudinal case series similarly produced positive outcomes, showing repeat dosing of ketamine to be moderately effective in four patients diagnosed with severe and enduring AN-R (*n* = 2) or EDNOS-BP (*n* = 2) and comorbid TRD of 11.0 years ± 1.4 [137]. Patients had previously completed partial hospitalization programs for their ED, reported persistent negative affectivity, and failed several trials of monotherapy antidepressants of adequate dose and duration. Ketamine was administered intramuscularly and/or intravenously at 0.5 mg/kg over 30–90 min, with subsequent doses titrated to 0.8–0.9 mg/kg depending on treatment toleration and response. Repeat dosing was scheduled at 4–6-week intervals spanning 12+ months, resulting in clinically meaningful changes in depression, as well as modest changes in anxiety and disordered eating. Interestingly, patients with AN-R demonstrated robust and sustained responses, compared to their EDNOS-BP counterparts, in addition to marked improvements in psychosocial functioning and quality of life trajectories. The differential degree of ketamine efficacy between ED subgroups merits further investigation.

Three case studies have also evaluated the effect of ketamine on ED symptomatology. Scolnick et al. published the first report of clinical remission in a 29-year-old woman with severe and enduring AN-R of 15 years, plus major depression and intermittent alcohol dependence, following repeated ketamine infusions adjunct to a ketogenic diet [138]. After adopting the high-fat, low-carbohydrate regimen for three months, the patient received four ketamine infusions starting at 0.75 mg/kg over 45 min, which were titrated to 1.0 mg/kg, 1.1 mg/kg, and 1.2 mg/kg for each subsequent dosing, respectively. Ketamine infusions spanned 14 days at unspecified intervals and were preceded by 4 g of sublingual ondansetron, a commonly used antiemetic, to prevent ketamine-associated nausea and vomiting. After the fourth dosing, the patient showed significant reductions in ED-related obsessive–compulsive tendencies and depression, with accompanying weight restoration. This led to complete and sustained recovery of cognitive and behavioral symptoms for six months post-treatment. The adjunctive therapy was also useful for managing her periodic alcohol dependence. Considering data that suggest metabolic dysregulation underlies and accelerates AN etiopathogenesis [144,145,146], the investigators queried whether the increase in ketone body production primed the response to ketamine.

Another case of severe and enduring AN complicated by major depression showed an initial but not sustained response to ketamine treatment [139]. The 29-year-old female presented with chronic refractory AN-R and comorbid MDD of 11+ years, with persistent borderline and narcissistic personality features, as well as active suicidality. Having failed several monotherapy and polytherapy medications, including standard and atypical antidepressants and antipsychotics, in addition to bilateral ECT, the patient underwent nine ketamine infusions. Dosing was administered at 0.5 mg/kg over 40 min scheduled twice weekly for 4 weeks, excluding the ninth infusion that occurred in isolation. Following infusion three, the patient progressed from active to passive suicidal ideation, experiencing complete remission after infusion eight. However, the response to ketamine rapidly diminished, resulting in an acute relapse of suicidality following infusion nine. While treatment efficacy was limited by ketamine’s short duration of effect, initial symptom improvement was significant and achieved faster than with prior ECT. Most recently, Ragnhildstveit et al. evaluated the use of ketamine-assisted psychotherapy (KAP) in treating a 21-year-old woman with extreme and enduring BN-BP of nine years [140]. This was the first ED report to administer ketamine with a psychotherapeutic component, and additionally for an “extreme” BN severity specifier, according to DSM-5 criterion (≥14 episodes/week) [147]. Upon clinic admission, the patient reported binge eating and purging by self-induced vomiting 40 times daily, despite care at the outpatient, inpatient, and residential level. In the patient’s final attempt at recovery, she underwent three courses of repeated KAP, with each course consisting of six sessions scheduled twice weekly at 48 h intervals (18 sessions total). KAP sessions comprised 30 min of preparatory psychotherapy, 40 min of intravenous ketamine (0.5 mg/kg), combined with guided psychotherapy, and 30 min of integration psychotherapy. A client-centered, humanistic approach to therapy was specifically leveraged to facilitate the process of self-actualization and behavior change. The patient progressively reduced her ED complaints and psychopathology over the treatment period, achieving complete cessation at three months post-treatment that sustained for over one year; an unexpected yet remarkable outcome given her initial chronic refractory state and extreme presentation.

## 3. Future Perspectives and Directions

Over the past two decades, ketamine has emerged as a novel treatment for refractory depression, exerting rapid (e.g., 2–24 h) and robust (e.g., *d* = 0.9–1.2) antidepressant activity [148,149]. Interest in transdiagnostic applications has since grown, with more than 140 clinical trials in the National Institute of Health (NIH) database (ClinicalTrials.gov, accessed on 5 February 2022) registered to investigate ketamine’s therapeutic potential. These trials specifically comprise major depression (98; 70%), suicidal ideation (21; 15%), post-traumatic stress disorder (7; 5%), obsessive–compulsive disorder (5; 3.6%), autism spectrum disorder (2; 1.4%), generalized anxiety disorder (1; 0.7%), borderline personality disorder (1; 0.7%), cognitive impairment (1; 0.7%), and schizophrenia (1; 0.7%) [128]. While data concerning ketamine and EDs are insufficient, preliminary, and by and large limited to AN over other subgroups [136,137,138,139,140], results are nonetheless encouraging. A recent mixed methods study (*N* = 200), examining patient attitudes toward complementary and emerging treatments for EDs, also provides supporting evidence for psychedelics, including ketamine. “I think everybody responds different to all sorts of treatment for eating disorders. I think it is essential to be open minded in regard to treatment” [150]. Importantly, patients expressed concern over psychedelics regarding safety, therapeutic setting, and trust among medical stakeholders, which they stated could be remedied through a priori education, controlled monitoring, and routine follow-up. Another qualitative study (*N* = 13) explored participant experiences with ceremonial ayahuasca and conventional therapy for AN and BN [151]. Thematic analysis revealed that ayahuasca produced rapid reductions in ED cognitions and behavior, allowed for painful memories and associated emotions to be processed, and catalyzed transcendent aspects of healing. 3,4-methylenedioxymethamphetamine-assisted therapy (MDMA-AT) has demonstrated similar positive outcomes. As part of a phase III, double-blind, placebo-controlled trial (*N* = 89), adults with severe PTSD meeting clinical or at-risk criteria for an ED diagnosis significantly decreased ED psychopathology, following MDMA-AT compared to therapy with placebo [152]. Results further showed that participants with greater ED symptoms experienced greater improvement at follow-up. Finally, Springs et al. investigated acute psychological effects of psychedelics in lifetime ED sufferers (*N* = 28), reporting clinically meaningful improvements in depression and well-being [153]. Taken together, and with the relevant literature on ketamine, these findings underscore the desire to develop and test psychedelic interventions for EDs, as well as their potential therapeutic value. Results additionally emphasize key methodological considerations in study design, which have already begun to inform ED study protocols [154] and ongoing trials (Identification No. NCT04714541 [ketamine], NCT04661514, NCT05035927, NCT04052568, NCT04505189 [psilocybin], NCT04454684 [MDMA], and NCT04878627 [cannabis]).

Future research should aim to investigate, establish, and optimize ketamine dose, duration, and frequency for EDs, in an effort to support clinical recommendations and evidence-based practice guidelines. Open-pilot studies and statistically powered feasibility, randomized, and implementation trials are therefore warranted. Moreover, empirically derived, standardized treatment regimens and outcome measures are suggested to facilitate comparisons across studies. Longitudinal assessments are additionally recommended to characterize clinical response for severe and enduring patients. Given that ketamine (1) may normalize glutamatergic dysfunction implicated in EDs [155,156], (2) promotes synaptogenesis and neuroplasticity [109,121,157,158] with a critical window following administration [159], and (3) has a short duration to relapse (2–6 weeks) [115], it is paramount ketamine be combined with psychotherapy moving forward, either adjunctively or concurrently. A growing body of literature has subsequently identified strategies to prolong its antidepressant effect, including KAP [160,161]. Ragnhildstveit et al. were the first to report on repeated KAP used to treat extreme and enduring BN, which utilized a person-centered, humanistic approach to psychotherapy, and resulted in complete and sustained remission [140]. The authors postulated that ketamine and psychotherapy act synergistically, with therapy augmenting the response to treatment and repeated sessions accounting for the durability of effect. Furthermore, the “emergence phenomena” of ketamine [162], characterized by euphoria, lucid dreams, and hallucinations, may facilitate therapeutic rapport, patient-provider bonding, and ultimately behavior change [163,164]. While treatment courses should permit flexible dosing and individualization, KAP generally follows a three-step model, consisting of preparatory psychotherapy (step 1), ketamine dosing (step 2), and integration psychotherapy (step 3). Discussing patients’ motives, intentions, and expectations for treatment, evaluating their current psychophysiological status, and providing a therapeutic environment conducive to the ketamine experience (i.e., “set and setting”) are essential factors to consider prior to treatment. Examining different psychotherapeutic approaches in this context is also recommended, which has progressively diversified over the years to include cognitive–behavioral [165], humanistic [140,166], functional–analytic [167], and somatic-based interactional [168] therapy.

## 4. Conclusions

The evidence presented here provides a conceptual, yet concise, summary of the use of ketamine in treating EDs. While the relevant literature remains small, studies signal therapeutic potential for this complex and largely under-researched population. In particular, ketamine may provide the greatest utility to clinical nonresponders, of whom are resistant to psychological, dietetic, and pharmacological interventions used in standard practice, and are prone to developing protracted ED pathology. Further research is necessary to explore the effects of ketamine on ED symptomatology and psychopathology, specifically across subgroups (critically in BN, BED, and AFRID) and diagnostic-dependent severity types (mild, moderate, severe, and extreme), as well as across the lifespan (from children to older adults). Data can then be leveraged to establish safety profiles, optimize dosing, and inform targeted treatment strategies at the individual patient level. Adjunctive and combination therapies—namely, KAP—also provide avenues for empirical investigation and for determining which contexts and interventional frameworks ketamine is best suited.

## Figures and Tables

**Table 1:** Characteristics and outcomes of studies using ketamine to treat eating disorders.

Study [Ref]	Design	Sample, Age/Mean Age	Diagnosis	Drug Regimen	Outcome
Dechant et al. [139]	Case study	*N* = 1, 29	AN-R + MDD	IV ketamine, 9 × 0.5 mg/kg over 40 min	Partial remission: depression and suicidality
Mills et al. [136]	Case series	*N* = 15, 33.3 years ± 6.5	AN-R, AN-BP	IV ketamine, 2–15 × 20 mg/h over 10 h	Partial remission: depression and OCD-related ED symptoms
Ragnhildstveit et al. [140]	Case study	*N* = 1, 21	BN-BP	IV ketamine, 18 × 0.5 mg/kg over 40 min	Complete and sustained remission: ED symptoms
Schwartz et al. [137]	Case series	*N* = 4, 36.8 years ± 8.4	AN-R + TRD, EDNOS-BP + TRD	IV/IM ketamine, 5–9 × 0.5 mg/kg titrated to 0.9 mg/kg over 30–90 min	Partial remission: depression, anxiety, and ED symptoms
Scolnick et al. [138]	Case study	*N* = 1, 29	AN-R + MDD	IV ketamine, 4 × 0.75 mg/kg titrated to 1.2 mg/kg over 45 min	Complete and sustained remission: depression and OCD-related ED symptoms

Notes: ED = eating disorder, AN-R = anorexia nervosa restricting subtype, AN-BP = anorexia nervosa binge/purge subtype, BN-BP = bulimia nervosa binge/purge subtype, EDNOS-BP = eating disorder not otherwise specified binge/purge subtype, MDD = major depressive disorder, TRD = treatment-resistant depression, OCD = obsessive–compulsive disorder, IV = intravenous and IM = intramuscular.
